# Acquired angioedema due to C1 inhibitor deficiency: real-world clinical characteristics and treatment outcomes

**DOI:** 10.3389/fimmu.2026.1836363

**Published:** 2026-05-22

**Authors:** İlkim Deniz Toprak, Simge Erdem, Pelin Korkmaz, Derya Unal, Osman Ozan Yegit, Merve Hormet Igde, Şule Çelik Kamacı, Okan Çetin, Nida Öztop, Semra Demir, Can Tüzer, Metban Mastanzade, Tuğrul Elverdi, Mehmet Emin Sezgin, Sevgi Kalayoğlu Beşışık, Aslı Gelincik

**Affiliations:** 1 Division of Immunology and Allergic Diseases, Department of Internal Medicine, Istanbul Faculty of Medicine, Istanbul Universitesi, İstanbul, Türkiye; 2 Division of Immunology and Allergic Diseases, TC Saglik Bakanligi Basaksehir Cam ve Sakura Sehir Hastanesi, İstanbul, Türkiye; 3 Division of Immunology and Allergic Diseases, TC Saglik Bakanligi Istanbul Sultan 2 Abdulhamid Han Egitim ve Arastirma Hastanesi, İstanbul, Türkiye; 4 Division of Hematology, Department of Internal Medicine, Istanbul Universitesi-Cerrahpasa Cerrahpasa Tip Fakultesi, İstanbul, Türkiye

**Keywords:** acquired angioedema, angioedema, autoimmunity, C1 inhibitor deficiency, lymphoproliferative disease

## Abstract

**Introduction:**

Acquired angioedema due to C1 inhibitor deficiency (AAE-C1INH) is a rare bradykinin-mediated condition that may mimic hereditary angioedema (HAE). Management is largely extrapolated from HAE data. Therefore, retrospective observations in AAE-C1INH are particularly valuable for informing future approaches. This study aimed to evaluate the clinical characteristics, underlying conditions, and treatment outcomes of patients with AAE-C1INH.

**Methods:**

We conducted a retrospective cohort study of adult patients diagnosed with AAE-C1INH, with a comprehensive review of demographic and clinical features.

**Results:**

Among 587 patients with recurrent angioedema, 1.7% had AAE-C1INH. Median onset age was 56.5 years (45.5–66.75), median follow-up was 60.5 months (23.25–66.5), and 20% were female. Clonal hematologic neoplasms were present in 60% of patients and monoclonal gammopathy of undetermined significance in 30%, with angioedema preceding the diagnosis of the underlying condition in 60% of cases. Long-term prophylaxis was required in 50% of patients. Antifibrinolytic agents showed limited efficacy, whereas attenuated androgens were associated with a marked reduction in attack frequency. Rituximab-based therapy effectively controlled angioedema, although relapse occurred during extended follow-up. Complete remission under Bruton’s tyrosine kinase (BTK) inhibition was observed despite persistently low complement levels; however, concurrent withdrawal of renin–angiotensin system blockers represents a potential confounder. Complement levels were observed to parallel treatment response in most patients, whereas this association was not observed in the patient receiving a BTK inhibitor.

**Conclusion:**

In this rare, well-characterized cohort with extended follow-up, angioedema frequently represented the earliest clinical manifestation of AAE-C1INH, preceding recognition of the underlying disorder. Antifibrinolytic prophylaxis showed limited benefit, whereas attenuated androgens were associated with reduced attack frequency. Furthermore, therapies targeting the underlying lymphoproliferative condition, including rituximab-based regimens and BTK inhibition, were associated with meaningful clinical benefit. These findings support an individualized, etiology-driven management approach and provide practical insights for clinicians managing this rare condition.

## Introduction

Angioedema (AE) is characterized by intermittent, localized, and self-limiting swelling of the subcutaneous and/or submucosal tissues resulting from increased vascular permeability and plasma extravasation. Recently, the DANCE consensus refined the classification of recurrent AE into five groups: mast cell–mediated, bradykinin-mediated, endothelial dysfunction–related, drug-induced, and AE of unknown cause (1). Within the bradykinin-mediated group, AE with C1 inhibitor (C1-INH) deficiency includes hereditary AE (HAE types I and II) and acquired AE due to C1-INH deficiency (AAE-C1INH). While HAE with C1-INH deficiency is a well-established and extensively studied disorder, AAE-C1INH represents a rare, non-hereditary subtype that can closely mimic HAE. It typically presents in adulthood and is frequently associated with lymphoproliferative disorders, monoclonal gammopathy of undetermined significance (MGUS), or autoimmune diseases (2). Clinical suspicion should arise in patients with late-onset AE and no family history of the disease (2).

The diagnostic workup includes measurement of serum C4 levels and assessment of both antigenic and functional C1-INH levels, followed by evaluation of C1q concentration (2, 3). In specialized settings, testing for C1-INH autoantibodies may be performed, and in selected cases, *SERPING1* gene analysis can help exclude hereditary forms (2, 4).

Although patients with AAE-C1INH share a similar clinical phenotype, the underlying aetiologies are heterogeneous. Current World Allergy Organization (WAO)/European Academy of Allergy and Clinical Immunology (EAACI) guidelines recommend that management should focus on identifying and treating the underlying cause. When this is not possible or remains insufficient, treatment strategies similar to those used in HAE could be implemented although it is important to emphasize that these approaches are off-label in this context (4).

Despite increasing recognition, the management of AAE-C1INH remains challenging due to its rarity, clinical heterogeneity, and the potentially life-threatening nature of attacks. The rarity of AAE-C1INH also limits the feasibility of randomized controlled trials, and current recommendations are based mainly on case reports, case series, and extrapolations from HAE data.

Although among available therapeutic options, rituximab has emerged as an effective disease-modifying treatment, particularly in patients with lymphoproliferative disorders or anti–C1-INH autoantibodies (5–9), evidence supporting standardized management strategies in AAE-C1INH remains limited.

Therefore, this study aimed to describe the clinical and demographic characteristics of patients with AAE-C1INH in a real-world referral cohort and to evaluate treatment responses.

## Methods

We conducted a retrospective review of the medical records of 587 adult patients (≥18 years) with recurrent AE attacks followed at the Department of Allergy and Immunology outpatient clinic in a tertiary referral center specialized in AE, after the ethics committee approval (3192364) was obtained. Written informed consent was obtained from all patients.

### Patient selection and data collection

Patients were included if they met one of the following criteria:

1. Low serum C4 and C1-INH levels or reduced functional activity, accompanied by decreased C1q levels
2. Low serum C4 and C1-INH levels or reduced functional activity, with normal C1q levels and a negative result for *SERPING1* gene mutation.

Exclusion criteria were having AE with an unexplained skin rash, attacks linked solely to food, drugs, or hymenoptera venom, or isolated non-recurrent episodes. In addition, patients in whom decreased C1q levels could not be demonstrated or in whom *SERPING1* gene mutation negativity could not be confirmed were excluded from the study. Patients with a history of AE in first-degree relatives were also excluded to minimize the likelihood of hereditary forms.

Demographic, clinical, and laboratory data, including treatment modalities and responses, were obtained from patient records, and current clinical status was verified through direct patient communication.

### Complement and genetic analyses

Laboratory data were obtained retrospectively from patient records. C1-INH protein levels had been measured by nephelometry, whereas functional C1-INH activity had been assessed using a coagulation-based assay. C4 levels had been determined by turbidimetry, and C1q levels had been measured by either radial immunodiffusion or nephelometry. *SERPING1* mutation analysis had been performed using Sanger sequencing.

### Treatment response criteria

Treatment response was defined based on changes in the frequency of AE attacks during follow-up compared with the pre-treatment baseline, with any occurrence of an AE attack considered an event regardless of the need for rescue medication or healthcare utilization. Treatment response was defined as a ≥50% reduction in monthly attack frequency compared with the pre-treatment baseline. Patients with a reduction of <50% or no change in attack frequency were considered non-responders (10).

For patients receiving therapy targeting the underlying disease, both clinical remission of AE and changes in complement parameters (C4 and C1-INH levels and/or function) were also taken into account when available. Patients experiencing one or more AE attacks per month were considered symptomatic, whereas those with no attacks during the specified follow up period were considered asymptomatic.

### Statistical analysis

All statistical analyses were performed using IBM SPSS Statistics version 23.

Given the relatively small sample size, analyses were primarily descriptive. Continuous variables were summarized using the median and interquartile range (IQR: 25–75), while categorical variables were presented as frequencies and percentages. Due to the limited number of cases, no subgroup comparisons or multivariate analyses were conducted.

The proportion of AAE-C1INH cases was calculated by dividing the number of identified cases by the total number of patients screened for recurrent AE. The HAE-to-AAE-C1INH ratio was determined based on the number of HAE and AAE-C1INH cases identified within the same cohort.

Figures were prepared using GraphPad Prism and Microsoft PowerPoint.

## Results

### Clinical and demographic characteristics of the patients

Among 587 patients screened for recurrent AE, 10 cases of AAE-C1INH were identified, corresponding to 1.7% of this selected referral cohort. The HAE-to-AAE-C1INH ratio was 14.4:1. One-fifth of AAE-C1INH patients were female (20%, n=2). The median age at AE onset was 56.5 years (IQR, 45.5–66.75), and the median age at diagnosis of the associated condition was 56 years (IQR, 43.75–68). Early-onset AE (before age 40) was observed in one of the patients. The median follow-up period was 60.5 months (IQR, 23.25–66.5).

The median duration of AE episodes was 72 hours (IQR, 66-75). The most frequently affected sites were the face (90%; lips 80%), followed by the abdomen (80%) and the extremities (70%). Other regions included the larynx (60%), eyelids (50%), tongue (60%), and genitalia (20%). Prodromal symptoms including irritability, weakness, nausea, and paresthesia occurred in 50% of patients (n=5), whereas erythema marginatum was absent. Trauma was the most common trigger (30%), with one patient (patient 9) experiencing attacks exclusively following trauma. Drug-induced AE was reported in two patients, one associated with an angiotensin converting enzyme (ACE) inhibitor (patient 8) and the other with an angiotensin II receptor blocker (ARB) (patient 10).

None of the patients reported a history of AE among first-degree relatives.

The conditions associated with AAE-C1INH were predominantly hematologic disorders, including chronic lymphocytic leukemia (CLL) (n=2), marginal zone lymphoma (MZL) (n=3), MGUS (n=3), and chronic myeloproliferative neoplasia (CMN) (n=1). The patient with CMN also had an associated autoimmune disorder, systemic lupus erythematosus (SLE). One patient declined further diagnostic evaluation.

Among the three MGUS cases, one patient each had IgA lambda, IgM kappa, and IgG kappa subtypes.

**Figure 1:**
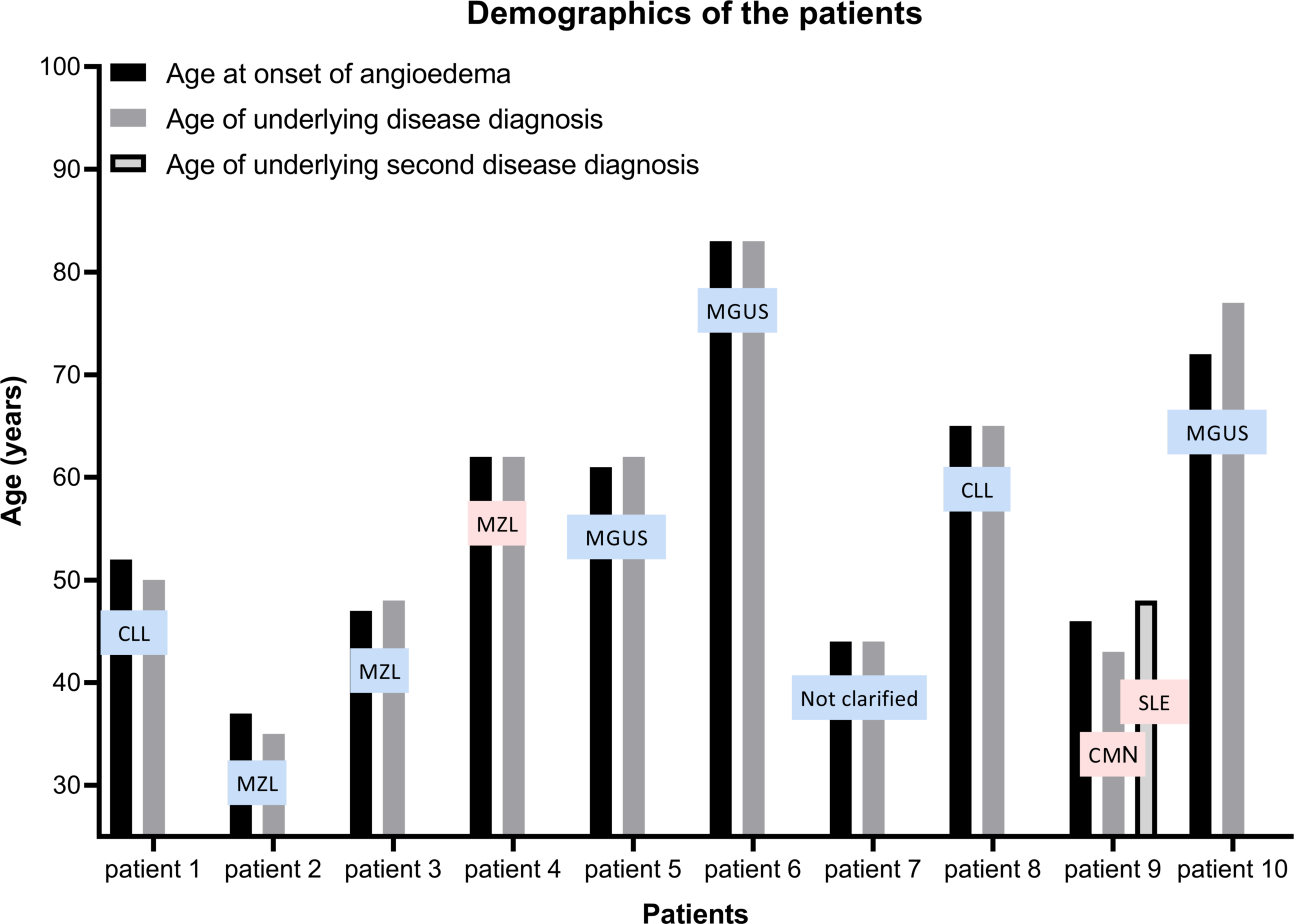
Demographic characteristics and clinical course of patients. Female patients are represented in pink and male patients in blue in the figure. SLE, systemic lupus erythematosus; CMN, chronic myeloproliferative neoplasm; CLL, chronic lymphocytic leukemia; MZL, marginal zone lymphoma; MGUS, monoclonal gammopathy of undetermined.

Median interval between AE diagnosis and the onset of hematological disease was 0 year [IQR: (−1)–2]. After exclusion of MGUS cases, the median interval was 1 year [IQR: (−0.25)–2.25]. In 30% of patients (one with CLL, one with MZL, and one with CMN concomitant with SLE), AE developed after the underlying disorder was diagnosed. In contrast, in 60% (three with MGUS, one with CLL, two with MZL), the systemic disease was identified following AE onset (**Figure 1**).

All three patients who first presented with AE prior to a diagnosis of a lymphoproliferative disorder (CLL n=1, MZL n=2) showed hematologic abnormalities including lymphocytosis, monocytosis, or anemia, on initial evaluation.

### Laboratory findings

All patients had low C4 levels. C1-INH levels were low in 9 of 10 patients (90%), while one showed normal levels with low functional activity. C1q levels were decreased in 7 patients (70%), whereas 3 patients had normal C1q despite low C4 and C1-INH levels. *SERPING1* mutation analysis, performed in 8 patients (80%), was negative in all cases (**Table 1**).

**Table 1:** Complement test results and genetic analyses of patients.

Patients	C4 level	C1-INH level	C1-INH function level	C1q level	*SERPING1* gene mutation	Complement profile after targeted treatment
Patient 1	▾	▾	NT	▾	NT	NT
Patient 2	▾	Normal	▾	▾	Negative	Normal
Patient 3	▾	▾	NT	▾	Negative	NT*
Patient 4	▾	▾	▾	Normal	Negative	Normal**
Patient 5	▾	▾	▾	▾	Negative	N/A
Patient 6	▾	▾	▾	▾	Negative	N/A
Patient 7	▾	▾	▾	Normal	Negative	N/A
Patient 8	▾	▾	NT	Normal	Negative	▾
Patient 9	▾	▾	▾	▾	Negative	▾
Patient 10	▾	▾	NT	▾	NT	N/A

*: It has been indicated as “NT” since no laboratory assessment was performed during the pre-relapse period under targeted therapy. **: It showed a secondary decline after disease recurrence. INH, inhibitor; NT, not tested; N/A, Not Applicable.

### Treatment of the angioedema attacks

#### Management of acute angioedema attacks

Six patients (60%) self-administered icatibant during attacks at standard doses recommended for HAE-C1INH, with favorable responses (4, 11). All patients had received C1-INH concentrate at standard HAE-C1INH dosing, which likewise yielding positive outcomes (4).

Fresh frozen plasma (FFP) was used in 6 patients (60%); however, one patient (Patient 7) showed no improvement after 2 FFP infusions for extremity AE and subsequently developed lip and oral mucosal swelling. The attack was controlled after administration of C1-INH concentrate at a dose of 1000 IU.

#### Short*-*term prophylaxis of angioedema

Short-term prophylaxis with C1-INH concentrate at a dose of 1000 IU was administered to 6 patients (60%) one hour prior to invasive diagnostic and/or therapeutic procedures, and no AE attacks were observed during or after these procedures. One patient underwent bone marrow biopsy without prophylaxis and experienced no complications, whereas another patient who did not receive prophylaxis prior to dental treatment developed uvular edema.

#### Long-term prophylaxis of angioedema

Long-term prophylaxis (LTP) was initiated in 50% of patients due to frequent AE attacks (**Table 2**). Regarding the choice of non-targeted LTP, these therapies were primarily preferred while diagnostic evaluation for an underlying condition was ongoing (patient 3), in cases where no indication for treatment of the underlying disease was identified (patients 3, 5 and 10), when treatment of the underlying disease did not result in remission (patient 9), or when no underlying disease could be identified (patient 7):

**Table 2:** Clinical outcomes of long-term prophylaxis strategies in angioedema.

Patient	Underlying disease	LTP regimen	Response	Treatment modification
3	MZL	Attenuated androgen 100 mg/day	Responder with ≈83% reduction (8–10 → 0–3 AE attacks/month)	Discontinued after disease-specific treatment
		Antifibrinolytic agent 1000 mg/day	Non-responder	Switched due to lack of response
		C1-INH concentrate 1000 IU/week	Responder with ≥75% reduction (8–10 → 2 AE attacks/month)	Final regimen
5	MGUS	Attenuated androgen 100 mg/day	Responder with 75% reduction (8→ 2 AE attacks/month)	Final regimen
7	Unknown	Antifibrinolytic agent 1000 mg/day	Non-responder	Switched due to lack of response
		Attenuated androgen 200 mg/day	Responder with 50% reduction (4 → 2 AE attacks/month)	Final regimen
9	CMN and SLE	Attenuated androgen 100–200 mg/day	Responder with ≥75% reduction (3–4 → 0–1 AE attacks/month)	Discontinued after control achieved with trauma avoidance measures
10	MGUS	Attenuated androgen 100 mg/day	Responder with ≈83% reduction 3/month → 1 every 2 months	Discontinued because of BPH-related symptoms

AE, angioedema; BPH, benign prostatic hyperplasia; CMN, chronic myeloproliferative neoplasm; LTP, long-term prophylaxis; MZL, marginal zone lymphoma; MGUS, monoclonal gammopathy of undetermined significance.

- Patient 3 (MZL) experienced 8–10 attacks per month during the diagnostic workup. Attenuated androgen therapy reduced the frequency to 0–3 attacks per month (83% reduction). Following treatment of the underlying disease, prophylaxis was discontinued. However, when attacks recurred seven months later due to relapse, antifibrinolytic therapy (1000 mg/day) was initiated but proved ineffective, and re-initiation of attenuated androgen therapy was not possible because of limited drug availability. The prophylaxis regimen was subsequently changed to weekly intravenous administration of C1-INH concentrate (1000 IU), which reduced attack frequency to 2–3 attacks per month, a 75% reduction.
- Patient 5 (MGUS) showed improvement with attenuated androgen therapy, with attacks decreasing from 8 to 2 per month (75% reduction).
- Patient 7 did not respond to antifibrinolytic therapy (1000 mg/day); switching to attenuated androgens reduced attack frequency from 4 to 2 per month (50% reduction).
- Patient 9 received attenuated androgens for six months, decreasing attacks from 3–4 per month to 0–1 per month (≥75% reduction). Prophylaxis was later discontinued when attacks were found to be exclusively trauma-induced and controlled by trauma avoidance.
- Patient 10 was treated with attenuated androgens, reducing attack frequency from 3 per month to 1 every two months (≈83% reduction). Therapy was discontinued after two years due to benign prostatic hyperplasia (BPH) requiring surgery.

### Treatment of the underlying disease

#### CLL patients

**Figure 2:**
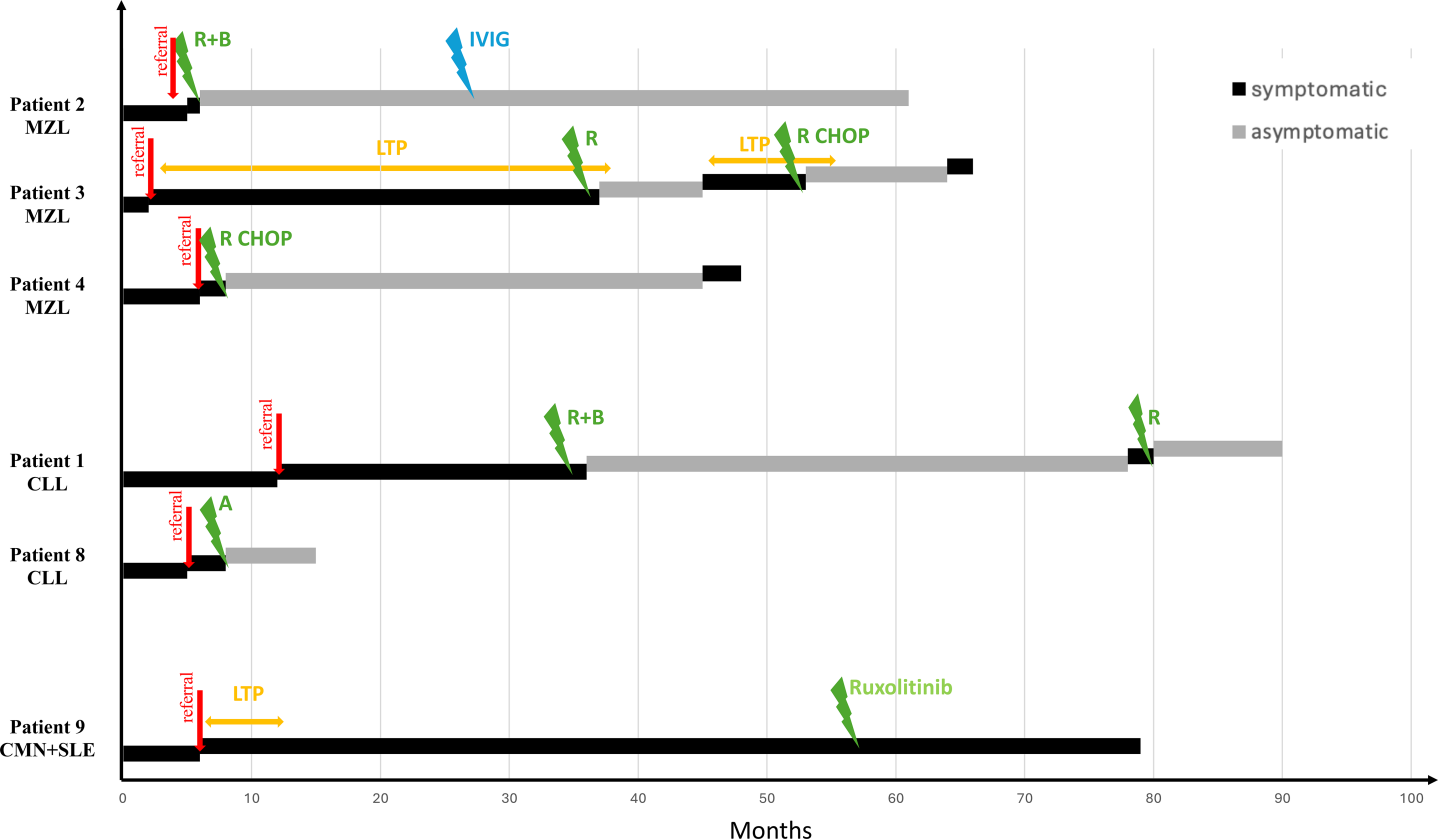
Response of Angioedema to underlying disease therapy. The red “referral” arrows indicate the patient’s presentations to the allergy clinic due to recurrent AE attacks. The green arrows represent treatments targeting the underlying disease, while the blue arrow denotes another therapy received in relation to the underlying condition. Patients are grouped by underlying diagnosis and ordered from top to bottom according to the frequency of these diagnoses; patient numbers reflect the previously assigned case numbers. A, Acalabrutinib; CLL, chronic lymphocytic leukemia; CMN, chronic myeloproliferative neoplasm; IVIG, intravenous immunoglobulin replacement therapy; LTP, long term prophylaxis; MZL, marginal zone lymphoma; R, rituximab; R+B, rituximab+bendamustine; R-CHOP: Rituximab, cyclophosphamide, doxorubicin, vincristine, and prednisone combination therapy.

Patient 1 with established CLL presented with AE attacks persisting for approximately 12 months prior to hematologic therapy (**Figure 2**). Combination therapy with rituximab and bendamustine was initiated 36 months after the first AE attack, resulting in a 42-month attack-free interval. Following AE recurrence, rituximab monotherapy induced a further 10 months of remission.

The other CLL patient, patient 8, was recently initiated on acalabrutinib, a Bruton’s tyrosine kinase (BTK) inhibitor, for recurrent AE attacks, including laryngeal involvement. Although CLL treatment was not otherwise indicated at that time, acalabrutinib therapy resulted in complete resolution of AE following the first dose, with no recurrence for seven months. Because discontinuation of an ACE inhibitor occurred concurrently, the relative contribution of each intervention could not be determined.

#### MZL patients

Among patients with MZL, patient 2 received rituximab and bendamustine, achieving durable remission with no AE recurrence over a 55-month follow-up period; however, due to the development of hypogammaglobulinemia, intravenous immunoglobulin (IVIG) therapy was initiated (**Figure 2**).

Patient 3 initially responded to rituximab monotherapy for MZL-associated hemolytic anemia, but experienced AE recurrence eight months after achieving hematologic remission. Subsequent R-CHOP (rituximab, cyclophosphamide, doxorubicin, vincristine, prednisone) therapy achieved an 11-month attack-free interval before AE and hematologic relapse occurred.

Patient 4, treated with R-CHOP at diagnosis due to B symptoms, remained AE-free for 36 months before recurrence involving the abdomen and eyelids, coinciding with decreased complement levels and hepatosplenomegaly consistent with MZL relapse.

#### MGUS patients

None of the three MGUS patients received disease-specific therapy. All continue to experience one to two AE attacks per month, primarily involving the extremities and lips, occasionally accompanied by abdominal pain.

#### Patient with concomitant CMN and SLE

Patient 9 had concomitant CMN and SLE. SLE developed five years after the initial CMN diagnosis, whereas AE attacks began four years after CMN diagnosis, occurring one year prior to SLE. At AE onset, she was receiving hydroxychloroquine and prednisolone for SLE and pegylated interferon for CMN. She continued both treatments for approximately five years. Following a period of loss to follow-up, she has been receiving ruxolitinib for the past two years. During this period, AE attacks were exclusively trauma-induced, without significant change in frequency during either CMN or SLE therapy.

#### Changes in complement levels with treatment of underlying disease

Complement levels were evaluated in relation to treatment response in four patients:

- In patient 2 (MZL), complement levels normalized following disease-specific therapy.
- In patient 4 (MZL), AE symptoms improved after the initial course of therapy, with subsequent normalization of C1-INH and later C4; however, both parameters declined again upon AE recurrence.
- In patient 8 (CLL) receiving acalabrutinib, C4 and C1-INH remained persistently low throughout a 7-month follow-up, despite complete clinical remission of AE symptoms.
- In patient 9 (CMN+SLE), complement levels remained persistently low despite disease-specific treatment, with ongoing AE attacks.

## Discussion

Our study has several notable strengths that enhance its clinical relevance and contribute meaningfully to the limited literature on AAE-C1INH. Despite the rarity of the condition, we provide detailed real-world data from a well-characterized cohort with a relatively long follow-up duration, allowing for a comprehensive evaluation of both clinical course and treatment responses. The inclusion of patients with diverse underlying conditions reflects real-life clinical heterogeneity and enhances the external validity of our findings. Building on this overall framework, several specific findings merit emphasis. First, the demographic and clinical profile of our patients was largely consistent with previous reports, particularly with respect to age at onset, attack localization, and the predominance of hematologic disorders as the underlying condition. Second, in the majority of cases, AE preceded the diagnosis of the associated systemic disorder, underscoring the importance of considering AAE-C1INH as a potential presenting manifestation of an occult hematologic disease. Third, among patients requiring LTP, antifibrinolytic therapy showed limited benefit in our cohort, whereas attenuated androgens were associated with a reduction in attack frequency. Fourth, serial complement measurements suggested that clinical remission may precede full biochemical normalization. Finally, rituximab-based regimens were associated with favorable clinical outcomes, and one patient treated with BTK inhibitor achieved complete clinical remission despite persistently low complement levels.

The estimated prevalence of AAE-C1INH is approximately 1 in 600,000 (3). Cicardi et al. reported a ratio of 10:1 for HAE to AAE-C1INH among patients with AE (2). In our referral population, AAE-C1INH accounted for 1.7% of adults evaluated for recurrent isolated AE, and the HAE-to-AAE-C1INH ratio was 14.4:1. The median age at onset in our cohort was 56.5 years, closely matching previous series, which consistently show that AAE-C1INH predominantly affects older adults (12–15). Although some prior studies deliberately excluded younger patients from their analyses (1, 8, 9, 16), our observation of a patient with disease onset before the age of 40 supports previous reports indicating that, although uncommon, earlier onset can occur (14, 15, 17). Notably, Bork et al. also reported that patients younger than 40 years had a higher prevalence of anti–C1-INH autoantibodies and MGUS (15). Within our series, the underlying condition in the patient with early-onset disease was MZL. Taken together, these findings emphasize that although AAE-C1INH typically presents in older adults, it may also be seen in younger individuals and should therefore be considered in the differential diagnosis regardless of patient age.

Similarly, the predominance of hematologic disorders in our cohort is consistent with prior studies identifying lymphoproliferative disease and MGUS as the most frequent associated conditions in AAE-C1INH (7, 14, 18, 19). Moreover, in the majority of cases, AE preceded the diagnosis of the associated disorder, with 60% of underlying conditions identified only after AE onset, in line with previous reports. Recognizing this temporal relationship is clinically important, particularly for hematologists and rheumatologists, as AAE-C1INH should prompt evaluation for an underlying disorder and may facilitate earlier diagnosis (10, 20). The clinical pattern of attacks in our patients, particularly the predominance of facial, abdominal, and extremity involvement, also parallels previous reports (8, 13, 17).

Beyond confirming known features of AAE-C1INH, our findings also provide clinically relevant observations regarding management. One notable finding was the limited benefit of antifibrinolytic therapy in patients requiring LTP. Current literature supports antifibrinolytic agents as an accepted first-line option for prophylaxis in AAE-C1INH (2, 21), with some studies reporting efficacy comparable to attenuated androgens (8, 14, 18). However, none of our patients achieved a meaningful response to tranexamic acid, whereas attenuated androgens appeared to reduce attack frequency in those who received them. This discrepancy likely reflects the marked heterogeneity of AAE-C1INH and reinforces the need for individualized treatment strategies rather than a uniform prophylactic approach. At the same time, this observation should be interpreted cautiously, as antifibrinolytic therapy was used at relatively low doses in our cohort, likely because of advanced age and comorbidities. Notably, attenuated androgens were also administered at modest doses, yet appeared to provide greater clinical benefit. Taken together, these findings may suggest differential treatment responsiveness across patients, but they are insufficient to support definitive comparative conclusions.

Another clinically relevant observation relates to complement monitoring. The diagnosis of AAE-C1INH has important clinical and therapeutic implications, particularly in the context of systemic autoimmunity and hematological malignancy. First, AAE-C1INH should be considered a potential clinical marker of an underlying disorder, as AE may precede the diagnosis of lymphoproliferative disease or autoimmune conditions, as also observed in our cohort. This highlights the need for systematic evaluation and longitudinal surveillance in affected patients.

In clinical practice, the presence of life-threatening AE attacks may warrant consideration as a potential criterion for initiating treatment of the underlying disease. Consistent with the literature, life-threatening attacks constitute a substantial proportion of cases, with reported rates of laryngeal involvement ranging from 15% to 63.3% in previous studies (7, 9, 10, 13). This consideration is further supported by the relatively high frequency of laryngeal involvement observed in our cohort (60%), underscoring the potential severity and life-threatening nature of AE attacks in this patient population.

Furthermore, even in the absence of overt AE symptoms, early assessment of complement parameters may facilitate earlier recognition of AAE-C1INH and contribute to more timely diagnosis, particularly in patients with lymphoproliferative and autoimmune diseases with an established association, where C4 may serve as a useful initial screening test.

Previous studies have also suggested that serial assessment of C4 and C1-INH activity may reflect disease control and that declining complement levels may herald relapse or progression of the underlying disorder (19, 22). Among our patients, recurrent AE episodes prompting hematologic reassessment were associated with relapse of the underlying hematologic disease and concomitant decreases in complement levels, supporting this relationship. Moreover, among patients receiving disease-specific therapy, normalization of complement parameters was observed in some cases. Notably, in one patient with available longitudinal data, clinical remission preceded full biochemical normalization, with C1-INH activity improving prior to C4 recovery. This observation suggests that clinical response may precede complement restoration and highlights the potential value of integrating both clinical and biochemical parameters in disease monitoring.

Our results also further support the importance of targeting the underlying disorder in AAE-C1INH. Rituximab is increasingly recognized as an effective therapeutic option, particularly in patients with lymphoproliferative disease, and recent reports suggest that its benefit may extend even to patients without an identifiable associated condition (5, 6, 8–10, 14, 20, 23–29). In this study, rituximab-based therapies were associated with favorable clinical responses. Moreover, both patients whose complement abnormalities failed to normalize had not received rituximab. In one of these cases, AE persisted, whereas in the other, acalabrutinib treatment was followed by complete clinical remission despite persistently low complement levels. Although this observation may suggest that rituximab contributes to both clinical and biochemical improvement, while BTK inhibition may control AE through additional complement-independent mechanisms, such interpretation must remain cautious. In the patient treated with acalabrutinib, concurrent ACE inhibitor exposure and subsequent discontinuation represent an important confounding factor, limiting attribution of the observed response solely to BTK inhibition. Nevertheless, this observation raises the possibility that suppression of B-cell receptor signalling and downstream activation of the malignant or autoreactive B-cell compartment may reduce the processes driving bradykinin generation, even before full biochemical normalization occurs. Furthermore, recurrence of AE after initially effective rituximab-based therapy was observed in patients with prolonged follow-up, consistent with prior reports indicating that benefit may diminish over time (16). This underscores that even when disease-specific therapy is effective, long-term monitoring remains essential.

Our cohort also highlights an important diagnostic consideration in patients receiving RAS blockers. Two patients developed AE episodes in association with ACE inhibitor or ARB exposure and were subsequently found to have AAE-C1INH. This finding is consistent with previous reports and emphasizes that C1-INH deficiency should be considered in patients presenting with recurrent bradykinin-mediated AE while taking these agents (8, 18, 30). Importantly, diagnostic evaluation should include both antigenic and functional C1-INH testing, as functional deficiency may not be detected from concentration measurements alone.

Several limitations should be acknowledged. First, the small sample size and single-center design substantially limit generalizability and preclude firm conclusions regarding treatment efficacy or comparative effectiveness. Second, the retrospective nature of the study resulted in incomplete data capture and limited standardization of attack assessment, treatment exposure, and follow-up intervals. In particular, treatment response was evaluated mainly on the basis of attack frequency, while attack severity, patient-reported outcomes, and quality-of-life measures could not be assessed reliably. Third, the heterogeneity of underlying disorders, concomitant medications, treatment regimens, and dosing strategies introduces substantial confounding, making it difficult to distinguish the independent effects of therapy. This is particularly relevant for the patient treated with acalabrutinib, in whom ACE inhibitor discontinuation occurred concurrently. In addition, the non-standardized and relatively low-dose use of antifibrinolytics and attenuated androgens limits the strength of any inference regarding their relative efficacy. Fourth, no formal comparative or inferential statistical analyses could be performed; therefore, the therapeutic observations presented here should be regarded as descriptive and hypothesis-generating. Finally, the absence of a control cohort consisting of patients with hematological disorders without AAE-C1INH limited our ability to determine whether the presence of AAE-C1INH influences the prognosis or clinical course of the underlying hematological disease.

Despite these limitations, this study contributes meaningful real-world data to a rare and clinically challenging condition for which high-quality evidence remains limited. Our findings support several practical messages: AAE-C1INH should prompt careful evaluation for an underlying hematologic disorder, particularly when AE occurs later in life; serial complement measurements may provide useful information for disease monitoring, although biochemical normalization may lag behind clinical improvement; and treatment response appears heterogeneous, underscoring the need for individualized management. Larger multicenter prospective studies with standardized outcome measures are needed to better define predictors of treatment response, clarify the role of complement monitoring, and optimize long-term therapeutic strategies in AAE-C1INH.

## Data Availability

The original contributions presented in the study are included in the article/supplementary material. Further inquiries can be directed to the corresponding author.
